# Apolipoproteins A and B and PCSK9: Nontraditional Cardiovascular Risk Factors in Chronic Kidney Disease and in End-Stage Renal Disease

**DOI:** 10.1155/2019/6906278

**Published:** 2019-12-14

**Authors:** Cristiana-Elena Vlad, Liliana Foia, Roxana Popescu, Iuliu Ivanov, Mihaela Catalina Luca, Carmen Delianu, Vasilica Toma, Cristian Statescu, Ciprian Rezus, Laura Florea

**Affiliations:** ^1^Department of Nephrology, “Dr. C. I. Parhon” Clinical Hospital Iasi, Iasi, Romania; ^2^Grigore T. Popa University of Medicine and Pharmacy, Iasi, Romania; ^3^Department of Cardiology, “Sf. Spiridon” Clinical Hospital Iasi, Iasi, Romania

## Abstract

**Purpose:**

Nontraditional cardiovascular risk factors as apolipoprotein A (ApoA), apolipoprotein B (ApoB), and the proprotein convertase subtilisin/kexin type 9 (PCSK9) increase the prevalence of cardiovascular mortality in chronic kidney disease (CKD) or in end-stage renal disease (ESRD) through quantitative alterations. This review is aimed at establishing the biomarker (ApoA, ApoB, and PCSK9) level variations in uremic patients, to identify the studies showing the association between these biomarkers and the development of cardiovascular events and to depict the therapeutic options to reduce cardiovascular risk in CKD and ESRD patients.

**Methods:**

We searched the electronic database of PubMed, Scopus, EBSCO, and Cochrane CENTRAL for studies evaluating apolipoproteins and PCSK9 in CKD and ESRD. Randomized controlled trials, observational studies (including case-control, prospective or retrospective cohort), and reviews/meta-analysis were included if reference was made to those keys and cardiovascular outcomes in CKD/ESRD.

**Results:**

18 studies met inclusion criteria. Serum ApoA-I has been significantly associated with the development of new cardiovascular event and with cardiovascular mortality in ESRD patients. ApoA-IV level was independently associated with maximum carotid intima-media thickness (cIMT) and was a predictor for sudden cardiac death. The ApoB/ApoA-I ratio represents a strong predictor for coronary artery calcifications, cardiovascular mortality, and myocardial infarction in CKD/ESRD. Plasma levels of PCSK9 were not associated with cardiovascular events in CKD patients.

**Conclusions:**

Although the “dyslipidemic status” in CKD/ESRD is not clearly depicted, due to different research findings, ApoA-I, ApoA-IV, and ApoB/ApoA-I ratio could be predictors of cardiovascular risk. Serum PCSK9 levels were not associated with the cardiovascular events in patients with CKD/ESRD. Probably in the future, the treatment of dyslipidemia in CKD/ESRD will be aimed at discovering new effective therapies on the action of these biomarkers.

## 1. Introduction

Worldwide, chronic kidney disease (CKD) represents a high public health priority [1]. Worldwide, over 2 million people require renal replacement therapy (hemodialysis (HD), peritoneal dialysis (PD), or kidney transplantation) to increase their survival rates [1, 2]. The prevalence of CKD has had an upward trend both in Europe and around the world, ESRD being merely the top of the iceberg [3]. CKD is an important cause of global mortality [1, 4]. The number of deaths caused by CKD has increased by 82.3% over the past two decades, being the third cause of the top 25 causes of deaths, after HIV/AIDS and diabetes [4].

Dyslipidemia in patients with impaired renal function is characterized by both qualitative changes in the cholesterol homeostasis and quantitative changes regarding the lipid parameters [5, 6]. Whereas in the general population dyslipidemia is described by the elevation of low-density lipoprotein cholesterol (LDL-C) [7], the progressive loss of renal function is associated with an increase of triglycerides, very low-density lipoprotein cholesterol (VLDL-C), and decreasing serum levels of the total cholesterol, HDL-C and LDL-C [5, 6].

**Figure 1:**
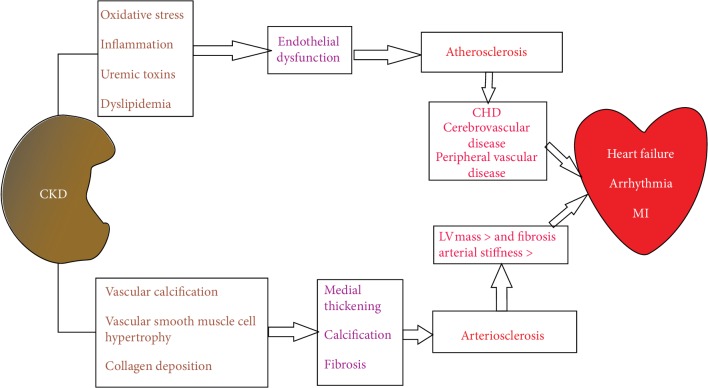
The pathophysiology of atherosclerosis and arteriosclerosis in patients with CKD. CHD: coronary heart disease; CKD: chronic kidney disease; LV: left ventricle; MI: myocardial infarction.

Cardiovascular mortality in dialysis patients is 10-20 times higher than that in the general population [1]. Cardiovascular death involves multiple pathogenic mechanisms: atherosclerosis, heart failure, and sudden death. Sudden death accounts for up to 25% of deaths from hemodialysis (HD) and occurs at the end of long-term HD and within the first 12 hours after HD [1]. Atherosclerosis and arteriosclerosis contribute to cardiovascular mortality in the general population [1, 8], while premature aging of the arteries, calcification, and arterial stiffness are characteristics of arteriosclerosis in chronic renal failure [1, 9]. Moreover, atherosclerosis affects arterial intima and is aggravated by CKD [1]. Several factors are involved in the pathogenesis of atherosclerosis and cardiovascular diseases: oxidative stress, inflammatory syndrome, malnutrition, arterial hypertension, endothelial dysfunction, vascular calcification, and dyslipidemia, both in the CKD and ESRD [7] (Figure 1).

The common biomarkers involved in the evaluation of the “dyslipidemic status” in the general population and CKD/ESRD patients are total cholesterol, LDL-C, HDL-C, and triglycerides, for the assessment of CVD risk. In addition, other possible biomarkers are represented by apolipoproteins (ApoA, ApoB, and ApoB/ApoA-I ratio) or PCSK9.

## 2. Objectives

This review proposes (1) to identify the studies showing biomarker level modifications (serum PCSK9, apolipoprotein A, and apolipoprotein B) in “uremic milieu” and (2) to depict current evidence of the association between these biomarkers and the development of cardiovascular events (stroke, heart failure, coronary pathology, and cardiovascular mortality) and (3) proposes new therapeutic approaches to reduce cardiovascular risk in CKD or ESRD patients.

## 3. Method: Search Strategy

We searched the electronic database of PubMed, Scopus, EBSCO, and the Register of Controlled Trials (Cochrane CENTRAL) from 3 January 2018 to 30 December 2018 for studies that evaluated the apolipoprotein profile in patients with CKD and ESRD and its cardiovascular outcomes. The terms used for searching were “apolipoprotein A-I”, “apolipoprotein A-IV”, “apolipoprotein B”, “apolipoprotein B/apolipoprotein A-I ratio”, “PCSK9”, “end-stage renal disease”, “ESRD”, “chronic kidney failure”, “CKD”, “advanced CKD”, “hemodialysis”, and “peritoneal dialysis”. Relevant references in these articles were searched manually to identify possible additional studies [10]. Randomized controlled trials, observational studies, including case-control studies, prospective or retrospective cohort studies, reviews, and meta-analyses were included if reference was made to apolipoproteins and their cardiovascular outcomes in CKD/ESRD [10]. Case reports were excluded, and studies were selected by two independent reviewers by screening the title and abstract. In a second phase, the full articles which conformed to the selection criteria were obtained, the essential data was extracted independently, and the results were analysed [10]. Discrepancies were resolved by discussion and consensus, and duplicates were excluded both manually and through a reference manager software [10]. Of these, only 18 met the inclusion criteria (Table 1). For the selected studies, we reviewed the full-text article and additional relevant publications were added after screening the reference section.

## 4. Results and Discussion

Apolipoproteins A and B and the ApoB/ApoA-I ratio are predictors of cardiovascular outcomes and potential biomarkers for cardiovascular mortality in both the general population and CKD/ESRD patients [11, 12]. It is not currently clear whether these biomarkers represent cardiovascular risk factors or could help in CVD diagnosis and the setting of the therapeutic targets in CKD/ESRD patients. In a comprehensive review, Vlad et al. showed that lipoprotein(a) (Lp(a)), the genetic polymorphisms of apolipoprotein(a), apolipoprotein E (ApoE), and apolipoprotein B (ApoB) undergo modifications in uremic patients, being correlated with cardiovascular events [10]. Furthermore, it was pointed out that in ESRD patients, Lp(a) levels were independent risk factors for atherothrombosis and cardiovascular mortality, LMW apo(a) phenotype was the best predictor for coronary events, single nucleotide polymorphisms in ApoE gene increased the risk of cardiovascular events, and ApoB had a significant correlation with the value of carotid intima-media thickness and vascular stiffness [10].

Our search has led to several studies with different results and conclusions (Table 1), which has created confusion regarding the roles ApoA-I, ApoA-IV, ApoB/ApoA-I ratio, and PCSK9 within the CKD/ESRD framework. This lack of consistency could be caused by the methodology of different types of studies (cross-sectional/case-control studies), small numbers of patients in the study groups, different clinical and laboratory outcomes, lack of homogeneous criteria for inclusion/exclusion, different definitions of endpoints, various periods of follow-up, or different statistical approaches.

## 5. Apolipoprotein A-I

### 5.1. Background

**Figure 2:**
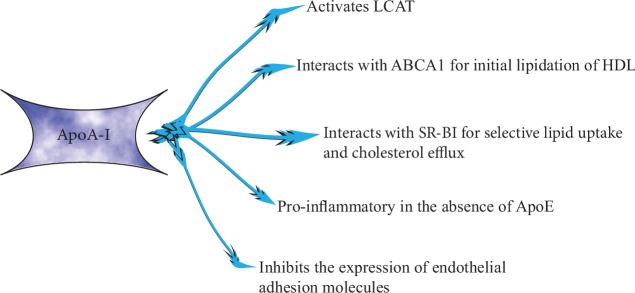
The biological functions of ApoA-I. In the liver, ApoA-I initiates the biogenesis of HDL and the lipid uptake and promotes cholesterol efflux. In the vascular endothelium, it maintains endothelial cell homeostasis. ApoE: apolipoprotein E; ABCA1: ATP-binding cassette transporters; LCAT: lecithin-cholesterol acyltransferase; SR-BI: scavenger receptor class B type I.

Apolipoprotein A-I (ApoA-I) is secreted predominantly by the liver and intestine as lipid-free ApoA-I and constitutes approximately 70% of HDL protein, being required for the normal HDL biosynthesis [13]. ApoA-I levels are strongly associated with those of HDL-C [11]. ApoA-I binds to circulating phospholipids and forms pre-*β* HDL (lipid-poor nascent discoid HDL particles) [7]. ApoA-I is involved in the elimination of excess cholesterol in tissues, which it incorporates into HDL for direct, indirect, or reverse transport via LDL to the liver [11]. ApoA-I inhibits the expression of endothelial adhesion molecules such as intercellular adhesion molecule-1 (ICAM-1) and vascular cell adhesion molecule-1 (VCAM-1), while it prevents the production of monocyte chemoattractant protein-1 (MCP-1), which are critical steps for the production of reactive oxygen species (ROS) in the arterial wall [7]. Likewise, ApoA-I has anti-inflammatory properties, which may contribute to its cardioprotective role [14]. In addition, ApoA-I displays strong antioxidant properties [7] as well as numerous functions (Figure 2) [15–17].

### 5.2. Apolipoprotein A-I in CKD and ESRD

ESRD is associated with a significant decrease in plasma ApoA-I and HDL-C [7, 18]. ApoA-I values in relation to ApoB values are used in estimating cardiovascular risk in patients with CKD/ESRD and CVD [19].

Thus, ESRD is associated with decreased levels of HDL-C and ApoA-I and may contribute to the atherogenic pathology [7]. In patients with CKD or ESRD, ApoA-I can efficiently evaluate the risk for cardiovascular disease [20], while its elevated level has been associated with a good survival rate [21].

### 5.3. Study Data

In a prospective cohort study conducted by Honda et al., the serum levels of ApoA-I were significantly decreased in patients with CKD 5D as compared to those with CKD stages 2-3 [22], in consent with the data reported by the ARIC study (Atherosclerosis Risk in Communities), in which CKD patients in stages 3-4 without coronary heart disease (CHD) had a low ApoA-I concentration [23].

#### 5.3.1. Pro Studies

In a cross-sectional multicenter study enrolling 995 HD patients, Hung et al. found that ApoA-I was positively correlated with total cholesterol, HDL-C, blood urea (BUN), and serum albumin [20], but ApoA-I had a negative correlation with pulse wave velocity (PWV) [24].

ApoA-I was negatively associated with cardiovascular morbidity in both predialyzed CKD patients (area under the curve (AUC) = 0.372; *p* < 0.0001) [21] and HD patients [11]. Moreover, ApoA-I has been significantly associated with the development of a new cardiovascular event [21] and has had the strongest independent correlation for CHD, the cut-off value for ApoA-I being 216.2 mg/dl [20]. Therefore, Zhan et al. also identified an association between ApoA-I and cardiovascular events in PD patients [25]. In addition, the low level of ApoA-I and serum creatinine constituted significant predictors of coronary pathology [20].

ApoA-I was significantly associated with all-cause mortality and cardiovascular mortality in HD patients [26] and PD patients [25]. In CKD patients, Cerezo et al. revealed that high ApoA-I concentrations have been significantly associated with the development of new cardiovascular episodes and were negatively associated with mortality (but with a lower level of significance) [21].

#### 5.3.2. Con Studies

Despite these findings, in an observational study that enrolled 412 HD patients, Honda et al. established that ApoA-I was not a risk factor for cardiovascular events [27]. Likewise, ApoA-I was correlated with cardiovascular events, but without any predictive strength in CKD patients (stages 2-5D) [22]. In an observational study with 91 HD patients, Bevc et al. revealed that ApoA-I did not correlate with carotid intima-media thickness (cIMT) [28]. Furthermore, in cohort CARE FOR HOMe (Cardiovascular and Renal Outcome in CKD 2–4 Patients—The Forth Homburg evaluation), which enrolled 443 patients, Rogacev et al. have revealed in a multivariate analysis after adjusting for confounders that ApoA-I levels were not associated with CV events (*p* = 0.483) [6].

## 6. Apolipoprotein A-IV

### 6.1. Background

**Figure 3:**
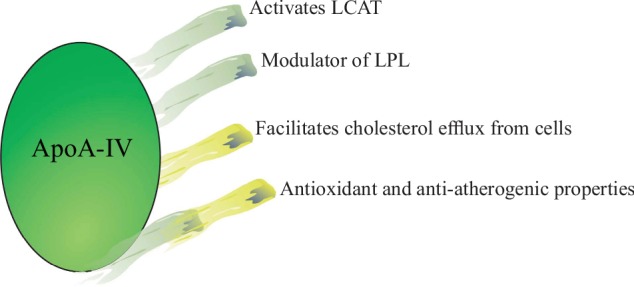
The roles of ApoA-IV. ApoA-IV has antioxidant and antiatherogenic functions. ApoA-IV activates LCAT and modulates LPL activation, favoring cholesteryl ester transfer from HDL to LDL. LCAT: lecithin-cholesterol acyltransferase; LPL: lipoprotein lipase.

Apolipoprotein A-IV (ApoA-IV) is a 46 kDa glycoprotein produced exclusively in the small intestine enterocytes during fat absorption and released into the lymph from the mesenteric duct, being incorporated into nascent chylomicrons [29–32]. In fasting plasma, ApoA-IV circulates as part of a lipid-poor, small HDL-like particle and ApoA-I free particles [29, 33]. ApoA-IV activates lecithin-cholesterol acyltransferase (LCAT) and modulates lipoprotein lipase (LPL) activation, favoring cholesteryl ester transfer from HDL to LDL, hence suggesting that ApoA-IV may behave like an antiatherogenic factor [29, 32]. ApoA-IV has antioxidant and antiatherogenic properties, and therefore, low levels of ApoA-IV increase the risk of CHD [31, 32] (Figure 3).

### 6.2. Apolipoprotein A-IV in CKD and ESRD

ApoA-IV also plays a relevant role in the reverse cholesterol transport [30–34], which is affected in patients with CKD [34]. Few studies have investigated serum ApoA-IV in patients with CKD and have shown that the kidney plays a crucial part in its metabolism [30, 32]. Renal function parameters (GFR, creatinine, and BUN) were the most important determinants of serum ApoA-IV levels in patients with CKD [29]. Immunohistochemical studies have indicated that ApoA-IV is filtered in the glomerulus and is mostly reabsorbed by proximal tubular cells [33, 34]. ApoA-IV is significantly raised in HD and DP patients [29, 30, 32].

ApoA-IV begins to grow from the initial stages of CKD, becoming thus an early marker of renal failure [29]. Serum ApoA-IV is associated with the development of atherosclerotic lesions in HD patients and can be useful for estimating the cardiovascular risk [30]. Moreover, low levels of ApoA-IV have been validated as a risk predictor for all-cause mortality and sudden cardiac death, being adjusted by the nutritional status [31].

### 6.3. Study Data

ApoA-IV is a key link between the decreased GFR and the presence of cardiovascular events [29]. Kronenberg et al. revealed that ApoA-IV significantly increased with the decreasing glomerular filtration rate (GFR), especially in dialyzed patients [29]. Subjects with mild and moderate CKD [29] and ESRD (HD patients) [30], who developed atherosclerotic complications (carotid artery plaques and a low ankle-brachial index), had a decrease in ApoA-IV plasma concentrations [30] as compared to control participants (24.9 ± 8.7 versus 22.3 ± 7.7 mg/dl, *p* < 0.15 for mild-moderate CKD) [29]. Also, ApoA-IV had a 60% sensitivity, a 69% specificity, and a cut-off value of 321.92 *μ*g/ml [30]. In addition, in a post hoc analysis performed in the Die Deutsche Diabetes Dialyse Studie (Study 4D), the scientists found the average ApoA-IV concentration of 49.8 ± 14.2 mg/dl about three times higher in HD patients than in the general population, which could not be influenced by statin administration [31].

#### 6.3.1. Pro Studies

Patients with elevated ApoA-IV levels displayed a lower risk factor on coronary atherosclerosis and cardiovascular disease as compared to low ApoA-IV subjects [30]. In the post hoc 4D study, at baseline, ApoA-IV concentrations were closely associated with the congestive heart failure and with ECG changes (arrhythmia, atrial fibrillation, atrial flutter, left bundle branch block, or right bundle branch block) [31]. Kronenberg et al. identified that ApoA-IV was associated with atherosclerotic complications and each ApoA-IV increase of 1 mg/dl decreased odds ratio by 8% for these complications (*p* < 0.011) [29]. Likewise, Omori et al. indicated that the ApoA-IV level was independently associated with maximum carotid intima-media thickness (cIMT) and cardiovascular disease [30].

Furthermore, Luczak et al. have carried out a comparative proteomic analysis of plasma proteins isolated from 75 patients in different stages of renal disease, 25 patients with advanced cardiovascular disease, and 25 healthy volunteers [35]. A direct comparison between CKD and CVD groups revealed significant differences in the accumulation of 2 proteins: ApoA-IV and *α*-1-microglobulin [35]. These proteins individually contributed to the formation of atheroma plaque, yet the inflammatory process was more powerful in patients with CKD [35]. On the other hand, the stimulation or inhibition of ApoA-IV in CVD and CKD groups suggested differences in the cholesterol transport efficacy [35].

Following the body mass index (BMI) adjustment, in ESRD patients with BMI > 23 kg m^2^, ApoA-IV was associated with all-cause mortality, heart rate mortality, sudden cardiac death, cerebrovascular events, and cardiovascular disorders [31]. Also, Kollerits et al. showed that the increase of 10 mg/dl was associated with a reduced risk of death of 11% (*p* = 0.001) [31].

#### 6.3.2. Con Studies

Contrary to these findings, in the same prospective randomized controlled trial, Kollerits et al. revealed that ApoA-IV did not impact upon the atherogenic risk: fatal myocardial infarction (*p* = 0.11) and nonfatal myocardial infarction (*p* = 0.14), fatal or nonfatal stroke (*p* = 0.18), or cardiovascular interventions (*p* = 0.62) [31]. Moreover, ApoA-IV did not associate with the cerebrovascular disease (*p* = 0.61) [31].

## 7. Apolipoprotein B and ApoB/ApoA-I Ratio

### 7.1. Background

**Figure 4:**
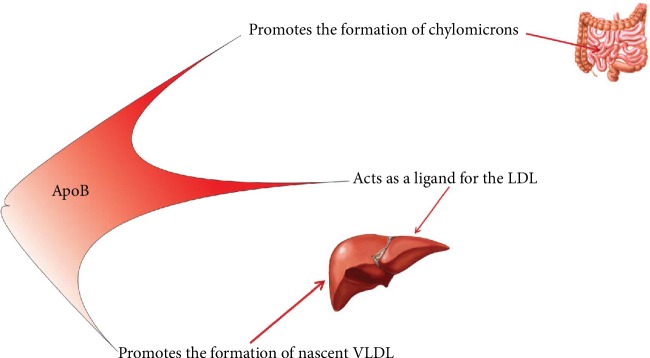
The roles of ApoB. In the liver, ApoB promotes the formation of nascent VLDL and also is essential for the linking of LDL particles to LDLR for cellular absorption and degradation of LDL particles. In the intestine, ApoB stimulates the formation of chylomicrons. LDL: low-density lipoprotein; VLDL: very low-density lipoprotein.

ApoB is a marker of dyslipidemia and is important in the binding of LDL particles to LDLR (receptor of LDL) for cellular absorption and degradation of LDL particles [11]. Apolipoprotein B (ApoB) is the primary protein component of very low-density lipoproteins (VLDL), intermediate-density lipoprotein (IDL), and LDL-C [11, 25, 26] and reflects the atherogenic particles from the body [11, 25, 26]. Other roles of ApoB are displayed in Figure 4 [36]. ApoB has two forms: ApoB-48 which is exclusively intestine secreted in chylomicrons and ApoB-100 which is only secreted by the liver in VLDL [37].

In the general population, several clinical studies (e.g., AMORIS (Apolipoprotein-related MOrtality RISk) [38] or INTERHEART [39]) have shown that the ApoB/ApoA-I ratio is strongly correlated with cardiovascular events such as myocardial infarction and stroke [40].

### 7.2. Apolipoprotein B and ApoB/ApoA-I Ratio in CKD and ESRD

Patients with CKD (G1-G4) have elevated ApoB values [23, 24]. In HD patients, ApoB concentration is within the normal range, in contrast with PD patients, who have elevated ApoB levels (due to overproduction) [41]. HD patients display advanced atherosclerosis that is associated with nontraditional risk factors (ApoB) [11, 28]. The reduction of ApoB could be a critical risk marker for eccentric left ventricular remodeling and could be useful for cardiovascular risk stratification in PD patients [42].

In CKD patients, the ApoB/ApoA-I ratio reflects the cholesterol balance between atherogenic and antiatherogenic lipoprotein particles [12], and an increased ratio highlights the progression of atherosclerosis [25]. Moreover, the ApoB/ApoA-I ratio represents a strong predictor for coronary artery calcifications [12] and myocardial infarction in CKD or ESRD [43]. Also, ApoB/ApoA-I ratio measurement was significantly associated with all-cause mortality and cardiovascular mortality in HD and PD patients [25, 26].

### 7.3. Study Data

In the ARIC study (Atherosclerosis Risk in Communities), patients with CKD stages 3-4 recorded an important ApoB/ApoA-I ratio increment as compared to those without CKD [23].

#### 7.3.1. Pro Studies

ApoB/ApoA-I ratio had a 100% sensitivity, a 77% specificity, and a cut-off value of 1.13 [11]. Besides, in the same case-control study conducted by Kirmizis et al., the ApoB/ApoA-I ratio was positively correlated with cardiovascular morbidity [11]. Moreover, in patients with stages 3-4 of CKD, the incidence of acute myocardial infarction was strongly associated with this ratio [43].

After adjustment for confounders, the ApoB/ApoA-I ratio was associated with cardiovascular events when serum levels exceeded the cut-off value, in patients with mild renal impairment [11], HD patients [27], and PD subjects [25]. Although the ApoB/ApoA-I ratio was associated with cardiovascular events, it displayed differences between the quartile models and the 1-SD increases, being influenced by interleukin-6 (IL-6) [27].

However, Sato et al. have found a substantial association of ApoB/ApoA-I ratio with all-cause and cardiovascular mortality in HD patients [26]. It appears that this ratio was significantly associated with all-cause mortality in PD patients [25].

#### 7.3.2. Con Studies

In contrast, in an observational study, Kim et al. reported that ApoB/ApoA-I ratio was not associated with coronary artery calcification in patients with mild renal impairment [12]. Moreover, the ARIC study did not detect an association between the ApoB/ApoA-I ratio and the risk for coronary disease [23].

## 8. Proprotein Convertase Subtilisin/Kexin Type 9 (PCSK9)

### 8.1. Background

Proprotein convertase subtilisin/kexin type 9 (PCSK9) is a serine protease of the subtilase family [44, 45], secreted primarily by the liver [44–46] and containing 692 amino acids [46]. PCSK9 is an enzyme accepted as a new biomarker for the lipid metabolism, a novel therapeutic target for hypercholesterolemia, because the inhibition of PCSK9 may be one of the options for lowering cardiovascular risk [47–49].

It triggers the reduction of LDLR levels without affecting the LDLR mRNA (messenger ribonucleic acid). PCSK9 determines the degradation of LDLR [50] and inhibits receptor recycling in the hepatocyte membrane [6]. The human PCSK9 protein (hPCSK9) is synthesized mainly in the liver, the kidney, the small intestine [46], and the brain [44, 45] via sterol regulatory element-binding protein 2 (SREBP-2) regulation [46]. Sterol regulatory element-binding proteins (SREBPs) coordinate the synthesis and cellular uptake for cholesterol and fatty acids [51]. SREBP-2 is primarily responsible for the activation of genes involved in the cholesterol synthesis, as opposed to fatty acid synthesis [51]. Thus, by activating the SREBP-2 pathway, statins increase the level of PCSK9, limiting the efficiency of these drugs for lowering LDL cholesterol [46].

**Figure 5:**
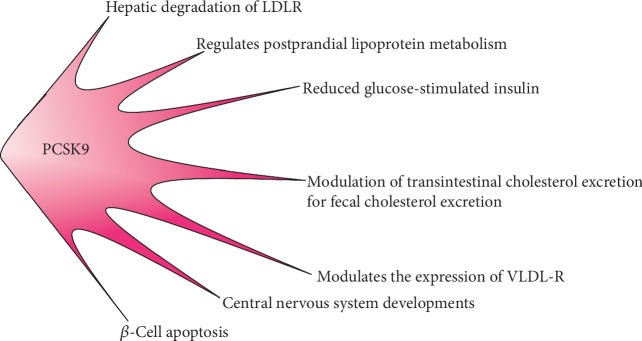
The biological functions of PCSK9. LDLR: low-density lipoprotein receptor; VLDLRs: very low-density lipoprotein receptors.

In the kidney, PCSK9 modulates the sodium absorption by degrading the epithelial sodium channel, and it also plays a part in the regulation of blood pressure [52]. PCSK9 has been identified in human pancreatic cells and does not modify endocrine pancreatic function [53]. Although adipocytes do not express PCSK9, they are rich in LDL and VLDL receptors, which play an important part in the hydrolysis of triglyceride-rich lipoproteins, and are helpful for fat storage in these cells [54]. In addition, PCSK9 is observed in carotid atherosclerotic lesions, especially in vascular smooth muscle cells [55]. Additional roles [56] are shown in Figure 5.

The human *PCSK9* gene is found on the human chromosome 1 and encodes the PCSK9 protein. The “gain-of-function” mutations of PCSK9 are associated with a rare form of autosomal dominant hypercholesterolemia (ADH), whereas “loss-of-function” mutations result in lowering cholesterol levels by reducing the CHD rate [46]. These genetic variants of *PCSK9* affect both the plasma concentrations of PCSK9 and the serum level of LDL-C [49], thus becoming new targets for the treatment of hypercholesterolemia [57].

## 9. PCSK9 and CKD/ESRD

In patients with CKD, the available evidence for PCSK9 is insufficient, with very few observational studies and with a small number of patients. At the same time, there are no data on the use of PCSK9 inhibitors to them [57].

In HD patients, PCSK9 levels were close to control group levels [58], or a differentiation of serum PCSK9 values before and after HD was not identified [59]. In PD patients, PCSK9 levels were close to those measured in patients with nephrotic syndrome [60].

### 9.1. Study Data

#### 9.1.1. Pro Studies

In a cross-sectional study comprising 134 diabetic patients with CKD, Elewa et al. identified that plasma PCSK9 was higher in patients with lipid lowering therapy, and the plasma PCSK9 values did not vary between patients with different eGFR or albuminuria categories [61]. During univariate analysis, Elewa et al. pointed out the significant positive correlation between the plasma PCSK9 level and the total iron binding capacity, vitamin E, renin, phosphaturia, and total serum cholesterol [61].

Also, Rogacev et al. have measured the serum level of PCSK9 in 2 independent cohorts: CARE FOR HOMe (Cardiovascular and Renal Outcome in CKD 2–4 Patients—The Forth Homburg evaluation), which included 443 patients, and the cohort LURIC (Ludwigshafen Risk and Cardiovascular Health Study) with 1450 patients [6], and they observed that plasma PCSK9 was poorly correlated with the total cholesterol, ApoB, and triglycerides [6].

#### 9.1.2. Con Studies

Conflicting results were further reported by Rogacev et al. who found no significant correlation between PCSK9 and GRF in nonstatin users of the LURIC cohort (*p* = 0.733) [6]. In addition, in the same two independent cohorts, Rogacev et al. observed that plasma PCSK9 values were correlated neither with baseline GFR values nor with LDL-C [6]. Likewise, plasma levels of PCSK9 were not associated with cardiovascular events in patients with low renal function [6].

Although PCSK9 is a potential determinant of serum cholesterol, no relationship with early or current cardiovascular disease has been identified, and thus, it cannot be considered a cardiovascular risk factor in CKD or ESRD patients [61]. Furthermore, Kaplan-Meier analysis revealed that serum PCSK9 levels did not predict cardiovascular events in any cohort (CARE FOR HOMe *p* = 0.622; LURIC *p* = 0.729) [6].

## 10. Therapy Options in CKD/ESRD for Apolipoprotein and PCSK9

Treating dyslipidemia with statins and ezetimibe results in favorable effects for cardiovascular disease (CVD) prevention, in patients with moderate CKD [57]. This therapeutic strategy has not proven effective in HD patients as indicated in the cardiovascular outcomes of 4D, AURORA, and SHARP studies [7]. Furthermore, in CKD/ESRD patients treated with statin, PCSK9 concentration was higher compared to nonstatin subjects [6, 59, 61].

In patients with CKD/ESRD, the use of PPAR*α* agonists is still controversial, and long-term safety and efficacy remain open to research [62]. By using niacin, besides having no additional benefit in patients with satisfactory control of LDL-C concentrations, tolerability was reduced [62]. ApoA-I mimetic is a new challenge for improving the lipid profile (in animal models), but clinical trials are still needed to confirm widespread use [62]. Although cholesteryl ester transfer protein (CETP) and cholesterol acyltransferase (ACAT) inhibitors have significantly improved HDL levels, results from major clinical trials have identified increased cardiovascular events [62].

Therefore, the need for an improvement of the lipid panel has emerged; new biomarkers (PCSK9) and new therapeutic strategies (monoclonal antibodies—evolocumab, alirocumab) are being further identified [57].

## 11. Key Points and Future Directions

The results of the studies focusing upon the association between apolipoproteins and CKD, after adjusting for cardiovascular events, suggest that supplementary mechanisms may be involved in addition to large vessel atherosclerosis. These include glomerulosclerosis, small vessel atherosclerosis, and direct toxic effects of apolipoproteins and lipids on the podocytes as well. The harmful effects are mediated by numerous mechanisms: expanded oxidative stress due to low levels of the apolipoprotein A1-enriched HDL fraction; the oxidized LDL-ApoB rich, local foam cell development; and the activation of inflammation [63].

The use of PCSK9 as a potential biomarker to identify patients with diabetic nephropathy who could benefit from anti-PCSK9 strategies and inhibition of PCSK9 could become an important treatment target in patients with CKD.

Patients with CKD should be enrolled in multicenter, randomized, double-blind trials (e.g., FOURIER, ODYSSEY) and closely monitored for the treatment efficacy against major cardiovascular events.

## 12. Conclusions

ESRD is associated with decreased levels of ApoA-I, with the increased levels ApoA-IV and ApoB, and with a high ApoB/ApoA-I ratio, but plasma PCSK9 levels are not associated with GFR decrease.

In patients with CKD/ESRD, even if there are the controversial results in the relationship between biomarkers and major cardiovascular events, ApoA-I, ApoA-IV, and ApoB/ApoA-I ratio are predictors of cardiovascular events. Nevertheless, these biomarkers could be useful for monitoring therapies with an impact upon cardiovascular morbimortality. Plasma PCSK9 levels are not associated with cardiovascular events in CKD or ESRD patients. However, circulating PSCK9 stands out as a promising biomarker for the diagnosis of dyslipidemia in patients with CVD and those affected by familial hypercholesterolemia.

In CKD patients, statins and ezetimibe can contribute to the prevention of CVD. The CETP, ACAT, and PCSK9 inhibitors have significantly improved HDL levels and reduced LDL-C. Also, ApoA-I mimetic is a new challenge for improving the lipid profile, but clinical trials are still needed to confirm widespread use.

Thus, in-depth studies are required on large cohorts of subjects along with the setting of clear targets of cardiovascular outcomes.

## Figures and Tables

**Table 1:** Characteristics of the included studies for cardiovascular outcomes.

Author	Study type	Apolipoprotein used	Outcomes	Population total	CKD patients	CKD stage	Dialysis type	Results
Kirmizis et al. [11]	Case-control study	ApoA-I ApoB/ApoA-I ratio	Cardiovascular morbidity	75	75	G5D	HD	(i) In the ROC curve analysis, serum ApoA-I was shown to be inferior as a marker of cardiovascular morbidity, with a likelihood ratio of 2.8 (ii) On logistic regression analysis, the age- and sex-adjusted OR for the presence of CVD was 2.0 (95% CI: 1.6 to 2.4), when ApoB/ApoA-I ratio values above 1.13 were compared with values below this cut-off point (iii) For ApoB/ApoA-I ratio values above 1.13, the OR did not change essentially after controlling for various confounders: nonlipid risk factors (OR = 2; 95% CI: 1.7-2.3), Lp(a) (OR = 2; 95% CI: 1.7-2.2), or markers of inflammation (OR = 1.9; 95% CI: 1.5-2.3)

Kim et al. [12]	Retrospective cross-sectional study	ApoB/ApoA-I ratio	Coronary artery calcification	7780	7780	G1-G3	—	(i) In multivariate logistic regression analysis, the ApoB/ApoA-I ratio was significantly associated with an increased risk of coronary artery calcification in participants with normal kidney function (OR = 2.411, 95% CI: 1.224-4.748, *p* = 0.011), while in the participants with mild renal insufficiency, the ApoB/ApoA-I ratio was not associated with coronary artery calcification (OR = 1.074, 95% CI: 0.395-2.925, *p* = 0.888)

Hung et al. [20]	Multicenter cross-sectional study	ApoA-I	Coronary heart disease	995	995	5D	HD	(i) Univariate analysis revealed that ApoA-I was associated with CHD (ii) Multivariate logistic regression analysis showed that ApoA-I was associated with CHD (OR = 3.27, 95% CI: 1.96–5.43, *p* < 0.01)

Cerezo et al. [21]	Prospective observational study	ApoA-I	New CV episodes	331	331	G3-G5	Predialyzed	(i) In the ROC curve analyses, the ApoA-I concentrations were negatively associated with mortality, but with a lower level of significance (area below the curve = 0.372; *p* < 0.0001) (ii) The only parameter that was significantly associated with the development of new CV episodes was the concentration of ApoA-I (area below the curve = 0.410; *p* = 0.035) (iii) In a multivariate Cox model adjusted by confounders, the risk ratio (RR) for each 10 mg/dl of ApoA-I was 0.915, with 95% confidence intervals (CI) of 0.844 and 0.992 (*p* = 0.031)

Honda et al. [22]	Prospective cohort study	ApoA-I	Composite cardiovascular events	111	111	G1-G5D	HD PD	(i) ApoA-I was associated with composite CVD events (HR = 2.86, 95% CI: 1.75-4.5, *p* = 0.0002) (ii) ApoA-I did not predict CVD events

Lamprea-Montealegre et al. [23]	Large multicenter cohort	ApoA-I ApoB/ApoA-I ratio	Risk of coronary heart disease	10137	1217	G1-G4	—	(i) CKD was associated with significantly higher concentrations of ApoB/ApoA-I ratios and significantly lower concentrations of ApoA-I (ii) ApoB/ApoA-I was associated with CHD risk (HR per one standard deviation = 1.22, 95% CI: 1.02-1.46)

Cicero et al. [24]	Cohort study	ApoA-I	Arterial stiffness	417	212	G2-G3	—	(i) In patients with CKD (G2-G3), the univariate analysis indicated that PWV was inversely related to ApoA-I (*p* < 0.05) (ii) In the stepwise multiple regression model that included all subjects (with normal function and CKD G2-G3), PWV was not associated with ApoA-I

Zhan et al. [25]	Retrospective cohort	ApoA-I ApoB/ApoA-I ratio	Cardiovascular events All-cause mortality	860	860	G5D	PD	(i) ApoA-I was correlated with all-cause mortality in model 2 (HR = 0.47, 95% CI: 0.25-0.89, *p* = 0.020) and model 3 (HR = 0.48, 95% CI: 0.24-0.94, *p* = 0.033) and with cardiovascular events in model 1 (HR = 0.47, 95% CI: 0.25-0.90, *p* = 0.022) and model 2 (HR = 0.39, 95% CI: 0.18-0.83, *p* = 0.015) (ii) In Cox regression analysis, after the adjustment in models, the ApoB/ApoA-I ratio was still associated with all-cause mortality (in model 3 HR = 1.60, 95% CI: 1.02-2.49, *p* = 0.040) and with cardiovascular events (in model 2: HR = 1.72, 95% CI: 1.05-2.81, *p* = 0.03 and in model 3: HR = 2.04, 95% CI: 1.21-3.44, *p* = 0.008)

Sato et al. [26]	Prospective cohort	ApoA-I ApoB/ApoA-I ratio	Cardiovascular disease- (CVD) related mortality	1081	1081	G5D	HD	(i) In the survival analyses, ApoA-I and the ApoB/ApoA-1 ratio were significantly related to all-cause and CVD-related mortality. Estimated survival curves by ApoA-I quartiles for all-cause and CVD-related mortality were significant (*p* = 0.001 and *p* = 0.001, respectively) (ii) In a multivariate Cox analysis, the ApoA-I (per 1-SD increase) was associated with all-cause mortality and CVD-related mortality (in model 2: HR = 0.75, 95% CI: 0.63–0.89, *p* = 0.001; HR = 0.77, 95% CI: 0.59–0.99, *p* = 0.04, respectively) (iii) In a multivariate Cox analysis, the ApoA-I (quartile IV versus quartile I) was associated with all-cause mortality and CVD-related mortality (in model 2: HR = 0.51, 95% CI: 0.32–0.81, *p* = 0.01; HR = 0.48, 95% CI: 0.24–0.98, *p* = 0.04, respectively) (iv) Survival curves by ApoB/ApoA-I ratio quartiles for all-cause and CVD-related mortality were significant (*p* = 0.001 and *p* = 0.02) (v) In a multivariate Cox analysis, the ApoB/ApoA-I ratio (per 1-SD increase) was associated with all-cause mortality and CVD-related mortality, even after adjustment in models (in model 3: HR = 1.16, 95% CI: 1.00–1.35, *p* = 0.046; HR = 1.38, 95% CI: 1.11–1.71, *p* = 0.004, respectively) (vi) In a multivariate Cox analysis, the ApoB/ApoA-I ratio (quartile IV versus quartile I) was associated with all-cause mortality and CVD-related mortality, even after adjustment in models (in model 3: HR = 1.65, 95% CI: 1.05–2.57, *p* = 0.03; HR = 2.56, 95% CI: 1.21–5.40, *p* = 0.01, respectively)

Honda et al. [27]	Prospective cohort study	ApoA-I ApoB/ApoA-I ratio	Death from all causes Composite CVD events	412	412	G5D	HD	(i) Quartiles of apolipoproteins were not associated with all-cause mortality (*p* > 0.05) (ii) Quartiles of ApoA-I were not associated with composite CVD events in models adjusted for age, sex, dialysis vintage, DM, history of CVD, and malnutrition (*p* > 0.05) (iii) ApoA-I was an independent risk factor in models adjusted for confounders including hs-CRP (HR = 0.62, 95% CI: 0.43-0.90, *p* < 0.05) (iv) Quartiles of ApoB/ApoA-I ratio was independently associated with CVD events in models adjusted with and without hs-CRP (HR = 2.21, 95% CI: 1.13-4.56, *p* < 0.05) and IL-6 (HR = 2.12, 95% CI: 1.09-4.33, *p* < 0.05) (v) Associations of apolipoproteins and ApoB/ApoA-I ratio with composite CVD events were also estimated in Cox hazards models of a 1-SD increase of variables (HR = 1.38, 95% CI: 1.04-1.85, *p* < 0.05) (vi) Each variable of ApoB/ApoA-I ratio was an independent biomarker of composite CVD events in this model adjusted for the time-varying covariates of HDL-C (HR = 5.80, 95% CI: 1.62-20.86, *p* < 0.05) and hs-CRP (HR = 5.52, 95% CI: 1.50-20.29, *p* < 0.05) (vii) The association of ApoB/ApoA-I ratio with composite CVD events disappeared when adjusted for IL-6 (*p* > 0.05)

Bevc et al. [28]	Observational study	ApoA-I	Asymptomatic atherosclerosis (IMT, plaque occurrence, and number of plaques)	91	91	G5D	HD	(i) Multiple linear regression analysis of nontraditional risk factors showed no relationship between ApoA-I values and IMTc (*p* > 0.05), plaque occurrence (*p* > 0.05), and the number of plaques (*p* > 0.05)

Kronenberg et al. [29]	Multicenter case-control study	ApoA-IV	Atherosclerotic complications	454	227	G1-G3	—	(i) In the logistic regression analysis, ApoA-IV emerged as a significant and independent predictor for the presence of atherosclerotic events (OR = 0.92, 95% CI: 0.86–0.98, *p* = 0.011)

Omori et al. [30]	Cross-sectional study	ApoA-IV	Cardiovascular disease Maximum cIMT	116	116	G5D	HD	(i) In a multivariable logistic regression analysis, after adjusting for confounders, high ApoA-IV concentration was associated with CVD and with maximum cIMT (OR = 0.24, 95% CI: 0.09–0.60, *p* < 0.005; OR = 0.33, 95% CI: 0.12–0.86, *p* < 0.05, respectively) (ii) In a stepwise multivariate regression analysis, A-IV concentrations were associated with maximum cIMT (*p* < 0.05) (iii) The serum ApoA-IV concentration was independently associated with maximum cIMT (adjusted *r*^2^ = 0.25)

Kollerits et al. [31]	Post hoc analysis of prospective, randomized, controlled trial 4D	ApoA-IV	Death from all causes Death from cardiac causes Combined cardiac events Combined cerebrovascular events Combined cardiovascular events	1224	1224	G5D	HD	(i) At baseline, ApoA-IV was inversely associated with the prevalence of congestive heart failure (OR = 0.81 per 10 mg dl^−1^ increment in ApoA-IV, *p* < 0.001) (ii) At baseline, ApoA-IV was correlated with ECG abnormalities such as arrhythmia, atrial fibrillation/flutter, and right or left bundle branch block (iii) At baseline, associations between ApoA-IV and variables reflecting atherosclerotic disease were weaker than those for congestive heart failure (iv) Each 10 mg dl^−1^ increase in ApoA-IV concentration was associated with an 11% reduced risk of death during the observation period (*p* = 0.001) (v) A significant association between ApoA-IV and all-cause mortality was found in the nonwasting group (HR = 0.89, 95% CI: 0.84–0.96, *p* = 0.001) (vi) In patients with BMI > 23 kg m^−2^, there was a relationship between ApoA-IV concentrations and death from cardiac causes (HR = 0.88, 95% CI: 0.80–0.98, *p* = 0.02), sudden cardiac death (HR = 0.83, 95% CI: 0.72–0.95, *p* = 0.006), and combined cerebrovascular events (HR = 0.84, 95% CI: 0.73–0.96, *p* = 0.01) (vii) Atherogenic events (fatal and nonfatal myocardial infarction or cardiovascular interventions), which were included in the overall group with cardiac events, were not associated with ApoA-IV concentration (HR = 0.98, 95% CI: 0.92–1.05, *p* = 0.62)

Luczak et al. [35]	Observational study	ApoA-IV	Formation of plaque	125	74	G1-G5	—	(i) CKD and CVD groups revealed accumulation of two proteins: ApoA-IV and *α*-1-microglobulin (ii) The results showed that at least two processes differentially contribute to the plaque formation in CKD- and CVD-mediated atherosclerosis (iii) The downregulation and upregulation of ApoA-IV in CVD and CKD groups suggested that substantial differences exist in the efficacy of cholesterol transport in both groups of patients

Holzmann et al. [43]	Large cohort	ApoB/ApoA-I ratio	Incidence of myocardial infarction	142394	142394	G1-G4	—	(i) The ratio of ApoB/ApoA-I was a strong predictor of myocardial infarction, both among subjects with and without renal dysfunction (HR = 3.35, 95% CI: 2.25–4.91 and HR = 2.88, 95% CI: 2.54–3.26, *p* < 0.05, respectively)

Rogacev et al. [6]	Cross-sectional observational CARE FOR HOMe Cross-sectional observational LURIC	PCSK9	(i) Acute myocardial infarction (ii) Surgical or interventional coronary/cerebrovascular/peripheral-arterial revascularization (iii) Stroke with symptoms > 24 hours (iv) Amputation above the ankle or death of any cause, cardiovascular death (v) Death immediately after intervention to treat CHD (vi) Fatal stroke (vii) Other causes of death due to CHD	443 1450	443 1450	G1-G4 G1-G4	—	(i) Kaplan-Meier analysis demonstrated no significant association between tertiles of PCSK9 and CV outcomes (*p* = 0.62). Separate analyses stratified by statin intake did not yield different results (statin users: *p* = 0.367; statin nonusers: *p* = 0.834) (ii) In multivariate analyses, we adjusted for confounders; PCSK9 was not an independent predictor of CV events (*p* = 0.206) (iii) In Kaplan-Meier analysis, tertiles of PCSK9 were not associated with cardiovascular deaths (*p* = 0.729). Separate analyses stratified by statin intake did not yield different results (no statin: *p* = 0.772; statin: *p* = 0.611)

Elewa et al. [61]	Cross-sectional observational study	PCSK9	Cardiovascular risk	134	134	G1-G4	—	(i) No relationship was observed between serum PCSK9 and cardiovascular risk

## Abbreviations

CKD: Chronic kidney disease
ESRD: End-stage renal disease
HD: Hemodialysis
PD: Peritoneal dialysis
ApoA: Apolipoprotein A
GFR: Glomerular filtration rate
PCSK9: Proprotein convertase subtilisin/kexin type 9
CVD: Cardiovascular disease
CHD: Coronary heart disease
cIMT: Carotid intima-media thickness.
